# The cost-effectiveness of a telephone-based intervention to support caregivers of older people discharged from hospital

**DOI:** 10.1186/s12877-019-1085-3

**Published:** 2019-03-04

**Authors:** David Youens, Richard Parsons, Christine Toye, Susan Slatyer, Samar Aoun, Keith D. Hill, Matthew Skinner, Sean Maher, Sue Davis, Rebecca Osseiran-Moisson, Rachael Moorin

**Affiliations:** 1 School of Public Health, Curtin University, GPO Box U1987, Perth, WA 6845 Australia; 2 School of Occupational Therapy, Social Work and Speech Pathology, Curtin University, GPO Box U1987, Perth, WA 6845 Australia; 3 School of Nursing, Midwifery and Paramedicine, Curtin University, GPO Box U1987, Perth, WA 6845 Australia; 4 Centre for Nursing Research, Sir Charles Gairdner Hospital, Hospital Ave, Nedlands, WA 6009 Australia; 5 School of Psychology and Public Health, La Trobe University, Bundoora, VIC Australia; 6 The Perron Institute for Neurological and Translational Science, Perth, WA Australia; 7 School of Physiotherapy and Exercise Science, Curtin University, GPO Box U1987, Perth, WA 6845 Australia; 8 Medical Division, Sir Charles Gairdner Hospital, Hospital Ave, Nedlands, WA 6009 Australia; 9 Corporate Nursing, Research & Education, Sir Charles Gairdner Hospital, Hospital Ave, Nedlands, WA 6009 Australia

**Keywords:** Caregivers, Family, Hospitals, Aged, Cost-effectiveness, Telephone based intervention

## Abstract

**Background:**

A telephone intervention for caregivers of older people discharged from hospital was shown to improve preparedness to care, reduce caregiver strain and caregiver distress. No cost-effectiveness analysis has been published on this, or similar interventions. The study aims addressed here were to examine whether positive outcomes for caregivers resulting from the Further Enabling Care at Home (FECH) program changed the use and costs of health services by patients; and to assess cost-effectiveness.

**Methods:**

A single-blind randomised controlled trial compared FECH to usual care. FECH involved a specially trained nurse addressing support needs of caregivers of older patients discharged from hospital. A minimum clinically important difference in preparedness to care was defined as an increase in Preparedness for Caregiving scale score of ≥ two points from baseline. Designated data collection was at: Time 1, within four days of discharge; Time 2, 15–21 days post-discharge; and Time 3, six weeks post-discharge. A last observation carried forward approach to loss to follow-up was used, with a sensitivity analysis including only those who completed all time points. Patient use of hospital, emergency department (ED) and ambulance services were captured for 12 weeks post-discharge using administrative data. Costs included nurse time supporting caregivers, resources used by the nurse, and time taken training the nurse to deliver FECH. Cost-effectiveness was assessed using decision trees for preparedness for caregiving.

**Results:**

Sixty-two intervention dyads and 79 controls provided complete data. A significantly greater proportion of intervention group caregivers reported improved preparedness to care to Time 2 (36.4% v 20.9%, *p* = 0.029), though this was not sustained to Time 3. The intervention cost $AUD268.28 above usual care per caregiver. No significant differences were observed in health service use between groups. The incremental cost-effectiveness ratio for each additional caregiver reporting improved preparedness to care at Time 2 was $AUD1,730.84.

**Conclusions:**

To our knowledge this is the first work to calculate the cost-effectiveness of a telephone-delivered intervention designed to support caregivers of older people post-discharge, and will support decision-making regarding implementation. Further research should examine different settings, and assess impacts on health service use with larger samples and a longer follow-up.

**Trial registration:**

Australian and New Zealand Clinical Trial Registry: ACTRN12614001174673. Registered 07/11/2014.

## Background

Chronic and co-morbid conditions commonly experienced by older people are often related to disability [1] meaning that needs for caregiving support can often be anticipated. Substantial caregiving input is often provided at home from family and friends (hereafter called ‘family caregivers’) and is becoming a common scenario associated with population ageing [2]. The value of family caregivers to the community is enormous in terms of monetary as well as social benefits, as documented in a recent Australian report [3]. Although the use of different methodologies to determine savings to the community from caregiver input means that comparisons need to be made with caution, estimates are that caregiver contributions to economies range from 0.3% of Gross Domestic Product (GDP) in France to 7.4% of GDP in the UK. Australian caregivers contributed 3.8% to the GDP in 2015, with an estimated monetary value of more than $AUD60 billion [3]. Although caregiving impacts vary substantially depending upon the unique context of the caregiving situation, poor caregiver health is frequently documented and has the potential to limit the sustainability of home care [4].

Unplanned hospitalisation occurs for older people in poor health for a variety of reasons including progression of existing illness, acute (new) health problems and problems with accessing appropriate care or support in the home [5]. A previous investigation by members of our team determined that caregivers of older patients discharged home from one acute medical assessment unit (MAU) felt underprepared to provide appropriate care after the discharge [6]. Although hospitalisation of the person receiving care can be experienced as a challenge to the caregiver [7], it also provides an opportunity for the hospital staff to determine and address needs for caregiving support [8]. If utilised in this way, the health of the caregiver may be maintained, unnecessary returns to hospital may be averted, and the long term sustainability of the home care situation enhanced.

We have previously published findings of a trial to determine the outcomes for family caregivers of older people from being included in an intervention – the Further Enabling Care at Home (FECH) program – upon discharge from hospital of the older person for whom they were providing care at home [8]. The FECH program is a telephone-administered intervention in which a specially trained nurse determines the caregiver’s understanding of the patient’s discharge letter, advising how to obtain further clarification if required, and facilitates the caregiver’s determination and prioritisation of their caregiving and support needs, providing guidance regarding access to support. This study found that caregivers included in the program experienced improved preparedness to care as well as reduced caregiver strain and reduced caregiver distress. Similar telephone-based programs have reported positive outcomes for caregivers of patients with various needs including stroke [9] and dementia [10]. Despite these findings, such interventions may be impractical or unappealing to policy-makers if the costs required to implement them are prohibitively high.

Given the continued increases in health-care expenditure in developed countries [11], cost-effectiveness analyses of interventions are increasingly important. Cost-effectiveness analysis can aid decision-makers in determining which interventions are worth funding by providing a better understanding of the investment required to achieve a certain outcome and therefore weighing the opportunity costs of funding one intervention as opposed to another [12]. Similarly, evidence on how an intervention may impact on the use of health services is useful to planners in deciding where funding should be allocated. That is, an intervention which results in reduced use of health services elsewhere in the system may be appealing as intervention costs may be partly or fully recovered through this reduction in use. To our knowledge, there are no published studies of telephone-based interventions to support caregivers of older patients which report on intervention costs, cost-effectiveness or on the potential health system impacts of interventions.

The aims of the current paper are therefore to (1) examine whether positive outcomes for caregivers resulting from FECH led to changes in the use and costs of hospital and ambulance services by patients; and (2) assess the cost-effectiveness of the intervention. We hypothesised that FECH would improve caregiver preparedness and that this would result in reduced use of health services by patients, partially offsetting the costs of delivering the intervention.

## Methods

The protocol for the trial, including the current cost-effectiveness assessment, was published previously [13]. Reporting follows the Consolidated Health Economic Reporting Standards (CHEERS) guidelines [14].

### Design

The study was conducted using a parallel group randomised controlled trial design and the study staff recruiting participants and collecting data were blinded to group assignment [8, 13]. Power calculations were based upon the primary outcome measure, preparedness to care as reported by family caregivers using the Preparedness for Caregiving Scale [15].

### Setting

Participants were recruited at point of discharge from an acute MAU in a tertiary metropolitan hospital in Perth, Western Australia, building on our previous work in this setting [6]. This hospital has more than 600 beds and the unit has 36 beds. Patients can only remain in the unit for up to 72 h so discharges and transfers occur at a rapid pace; at the time of the study the average length of stay was only 1.4 days.

### Participants

Patient-caregiver dyads were eligible for inclusion in the study when they comprised a patient aged 70 years or older being discharged home plus a family caregiver who could speak and read English. Although reaching the age of 65 has for many years triggered access to Australian aged care services, supports, and pensions, change is now starting to occur because of increased life expectancy. Recent planning documents use 70 as the definition of “older” [16] hence our study adopted this threshold. As defined by Aggar (2011), family members or friends providing care, support, and assistance without payment were considered to be “family caregivers” [17].

### Intervention and control conditions

The control condition was usual discharge care. Usual discharge care consisted of providing the patient with a copy of the discharge letter from the hospital doctor to the patient’s regular medical practitioner plus medications, prescriptions, referrals or outpatient appointments [8]. The intervention, the Further Enabling Care at Home program, is described in detail elsewhere [8, 13]. The central component of the program is the nurse’s inclusion of the caregiver in a caregiver-led conversation using the Caregiver Support Needs Assessment Tool (CSNAT) [18–20]. This tool is provided to the caregiver a few days before the phone conversation to facilitate reflection upon the questions, which are about the needs for support to (a) enable the caregiver to care for the patient and (b) address caregivers’ needs to look after themselves [19]. When the caregiver has identified, rated and prioritised up to three most pressing needs, the nurse guides access to the most urgently required support [19].

### Procedures

#### Recruitment and randomisation

Eligible (patient-caregiver) dyads were recruited [8]. Caregivers were required to provide written consent to participate. Patients provided written consent to study participation if their health and cognitive capacity allowed them to do so. When illness, cognitive impairment, or intellectual disability meant that the patient was unable to consider consenting to study participation, patient data (already collected routinely by the Department of Health) were included in this study under a waiver granted by the Human Research Ethics Committee of the Department of Health in accordance with the National Statement on Ethical Conduct in Research [21]. An ‘opt out form’ was provided for these patients in case there was a later opportunity – if and when their health improved – for them to consider inclusion of their data in the study, up until the time when the data were provided to the research team [13]. Each dyad was randomly allocated to either the control condition or to receive the intervention plus usual discharge care [8, 13]. Trial randomisation, recruitment and blinding have been described in more detail previously [8, 13].

#### Measures and data collection

Designated data collection time points for outcome measures were as follows: Time 1, within four days of discharge; Time 2, 15–21 days after discharge; and Time 3, six weeks after discharge [20]. Demographic data for patients and information about the patients’ health conditions and the caregiving situation were obtained from the caregivers at Time 1 [8, 13]. Time 1 data collection time points were always prior to intervention commencement for the experimental group. Similarly Time 2 data collection time points were always after intervention completion. This required careful management of the study protocol as outlined in a previous paper [8].

##### Caregiver outcomes

Caregiver outcomes included here are those found to differ significantly between groups in previously published analyses [8]. The primary caregiver outcome was an increase in preparedness to provide care at home at Time 2, as measured by the Preparedness for Caregiving Scale. This tool asks about caregivers’ preparedness for eight different aspects of caregiving, with five levels for each item (from 0, not at all well prepared, to 4, very well prepared). For this outcome we defined a minimum clinically important difference (MCID) in preparedness for caregiving as an increase in score of at least two points [8] (i.e. a two-point improvement on a single item or one-point improvement on two items). Secondary caregiver outcomes showing significant improvements for the intervention group (vs control) were the Family Appraisal of Caregiving Questionnaire for Palliative Care (FACQ-PC) [22] distress score at Time 2, and FACQ-PC strain score at Time 3. For these secondary outcomes there was no published MCID, hence statistical significance and effect sizes are reported for these outcomes.

We additionally report on caregiver self-rated health, rated using the SF12 Version 2 [23].

##### Patient outcomes

The main patient outcomes considered were time to, and length of, hospitalisation during the post-discharge (i.e., post-recruitment) period (minimum 3 months), as well as presentations to emergency departments (ED) and use of the ambulance service during that period. In the event that the patient died, the date of death was used to inform the analyses. Hospital, ED and mortality data were provided via the WA Data Linkage System [24]. These data include the date of service, reason for service, and triage code for ED presentations. Admission/separation dates, primary diagnosis and co-diagnoses, diagnostic related group, and mode of transport to hospital were obtained for hospitalisations. Dates of any deaths were established from mortality data [13]. Hospital services were costed on the basis of the diagnostic related group recorded and ED presentations on the basis of urgency related group, using the relevant cost weights reported in Independent Hospital Pricing Authority reports [25]. A cost of $AUD916 was applied to each ambulance service provision [26].

#### Intervention costs

A bottom-up approach was used for intervention costs. These consisted of the nurse time for each telephone contact including the phone call, time to implement and organise resources and write notes following the contact; stationery and postage costs; training of the FECH nurse including nurse and trainer time; development of a resource manual for the nurse to guide access to supports for caregivers; and telephone charges. The nurse recorded durations of all telephone contacts plus stationary and postage costs for each dyad hence these costs varied between dyads, whereas training costs, development of the resource manual and telephone charges were “fixed” and were divided equally across all dyads. All costs are in 2015 Australian dollars, which in 2015 was worth approximately US$0.73, €0.67 and £0.49 [27].

### Statistical analyses

As groups were balanced at baseline [8], differences in the outcomes between groups were tested without any adjustment for other variables.

#### Preparedness to care and secondary outcomes

The Chi-square statistic was used to compare the proportions of carers reporting an improved preparedness to care in excess of the MCID between the control and intervention groups.

Where a participant withdrew from the study, their missing data were imputed conservatively by assuming that their preparedness did not change from the previously assessed time-point, following a Last Observation Carried Forward (LOCF) approach. Two analyses were performed: firstly using the full dataset with the LOCF where necessary, and secondly using only participants who completed all follow-up data collections (“per-protocol” analysis).

Changes in secondary outcomes (caregiver strain, caregiver distress and self-rated health) were assessed as continuous variables. Between-group differences were tested using a mixed model, which included all 3 time points so that correlation between measures on the same participant could be taken into account.

#### Use of additional health services

A chi-square test was used to compare the proportion having health service contact (hospitalisation or ED presentation) between groups.

The Kaplan-Meier curve of the time (days) to first contact for each group was drawn and the Log-rank test used to compare curves for the two groups. For those admitted to hospital, total length of stay (including all admissions) was compared between groups using a non-parametric Wilcoxon 2-sample test. Total acute costs were compared between groups by applying a Box-Cox transformation to costs, and performing a t-test on transformed costs. The SPSS version 22 software [28] was used for the analyses, and, following convention, a *p*-value < 0.05 was taken to indicate a statistically significant association in all tests.

### Cost-effectiveness

Since only preparedness to care, caregiver strain and caregiver distress showed a statistically significant difference across arms of the trial, the cost effectiveness analysis was limited to these outcomes. Cost-effectiveness was calculated using decision trees constructed using TreeAge Pro 2017 [29]. For both the intervention and control branches there were two possible outcomes: an improvement at least equal to the MCID on the Preparedness for Caregiving Scale, or no improvement (including an improvement below the MCID). Cost-effectiveness was reported as an Incremental Cost-Effectiveness Ratio (ICER), that is, the cost of the intervention required for each additional caregiver reporting an improvement in preparedness to care, compared to usual care [30]. As the follow-up was short discounting was not applied to either outcomes or costs. Cost-effectiveness is reported from the perspective of the acute care system, since out of hospital costs (e.g. general practitioner and prescribed medications) were not included.

#### Secondary outcomes

ICERs were constructed for secondary outcomes (caregiver strain and caregiver distress) similar to the primary outcome. As the secondary outcomes were analysed in terms of mean changes, rather than as the proportion improving, the ICERs for these outcomes represent the cost for the equivalent of a one-point increase on each scale for one caregiver.

#### Deterministic sensitivity analysis

The following deterministic sensitivity analyses were performed:

1. “Per-protocol” analysis in which effectiveness was calculated based on completers only. The intervention costs of non-completers were distributed equally among completers to provide conservative estimates of intervention costs
2. Analyses in which nurse time delivering the intervention reflected the 25th and 75th percentiles observed
3. Analyses in which the intervention’s effectiveness was assumed to reflect the limits of the 95% confidence interval of the proportion of caregivers reporting an improvement in preparedness to care

#### Probabilistic sensitivity analysis

Probabilistic Sensitivity Analysis (PSA) examined the impacts of sampling uncertainty in intervention costs and effectiveness. A PSA with 1000 samples was run with variation in intervention costs, the proportion of improvers in the usual care group and the proportion of improvers in the intervention group. Variation was based on the mean costs, the proportions improving in each group and the standard errors on each of these measures.

## Results

### Participant numbers and characteristics

Participant numbers and characteristics have been reported in detail previously [8]. Briefly, of 583 dyads assessed for eligibility between April and November 2015, 77 intervention dyads were included in analysis (62 of whom completed all data collections) and 86 control dyads (79 of whom completed all data collections). At baseline the groups were balanced on all caregiver characteristics (e.g. demographics, relationship to patient, type of care provided) and on all outcome measures. Data collection time points changed from those designated in the protocol due to limited caregiver availability, however mean time periods between data collection time points did not significantly differ between groups [8]. The most common reasons for admission to the MAU were: Diseases of the musculoskeletal system and connective tissue (28.8% of participants), diseases of the circulatory system (25.8%), and endocrine, nutritional and metabolic diseases (20.9%). Other conditions each affected less than 10% of the participants.

### Caregiver outcomes

Results previously published showed that FECH was associated with significant improvement in preparedness to care, relative to the control group, measured on a continuous scale at both Time 2 and Time 3.

When categorised into those who did or did not achieve the 2-point change in preparedness, and using the conservative LOCF strategy to replace missing data, it was found that 36.4% of the intervention group reported an improvement to Time 2 compared to 20.9% of controls (*p* = 0.029, Table 1; odds ratio = 2.16). When records with missing data were excluded the difference between groups was greater, with 45.2% of the intervention group recording an improvement to Time 2 in comparison to 21.7% of the control group (*p* = 0.003, Table 1; odds ratio = 2.97). Analysis of the proportions improving from Time 1 to Time 3 showed no significant change for either analysis (LOCF: *p* = 0.302, per protocol: *p* = 0.076).

**Table 1:** Proportion of carers reporting improvement in preparedness to care equal to or greater than the MCID, by group

To time period (from baseline)	Dataset	Group	Improved n/N (%)	*p*-value
Follow-up 1	LOCF	Control	18/86 (20.9)	0.0288
		Intervention	28/77 (36.4)	
	Per protocol	Control	18/83 (21.7)	0.0027
		Intervention	28/62 (45.2)	
Follow-up 2	LOCF	Control	29/86 (33.7)	0.3019
		Intervention	32/77 (41.6)	
	Per protocol	Control	29/79 (36.7)	0.0762
		Intervention	32/62 (51.6)	

To Time 2, caregivers in the intervention group reported a mean reduction in distress of 0.24 points compared to 0.09 in the control group (*p* = 0.036). However, this difference did not persist to Time 3 (Table 2). Changes in strain scores did not differ between groups at Time 2, but at Time 3 had reduced by 0.15 in the intervention group compared to a 0.04 point increase in controls (*p* = 0.040) (Table 2).

**Table 2:** Changes in the FACQ strain and distress scales (based on LOCF analyses)

To time (from Time 1)	Variable	Control mean (SD)	Intervention mean (SD)	Effect size	*p*-value^#^
Time 2	Strain	0.05 (0.57)	− 0.11 (0.45)	0.30	0.0798
	Distress	−0.09 (0.65)	− 0.24 (0.46)	0.27	0.0354
	SF12 - Physical	−1.26 (8.05)	−0.11 (4.63)	0.17	0.5289
	SF12 - Mental	0.80 (7.82)	2.78 (8.72)	0.24	0.2049
Time 3	Strain	0.04 (0.65)	−0.15 (0.55)	0.32	0.0394
	Distress	−0.06 (0.52)	−0.17 (0.54)	0.21	0.0728
	SF12 - Physical	−1.23 (8.12)	−0.85 (6.31)	0.05	0.8596
	SF12 - Mental	1.12 (9.41)	1.39 (8.50)	0.03	0.7534

# *P*-values were obtained for each outcome using a Mixed model which included all 3 time points (rather than pairwise t-tests)

Changes in SF12v2 scores did not differ between groups at Time 2 or Time 3 (Table 2). We assessed changes in individual components as some domains assessed through the tool (e.g. mental health) may be more likely to show benefit from the intervention than others. Changes in scores did not differ between groups on any domain.

### Use of additional health services

**Fig. 1:**
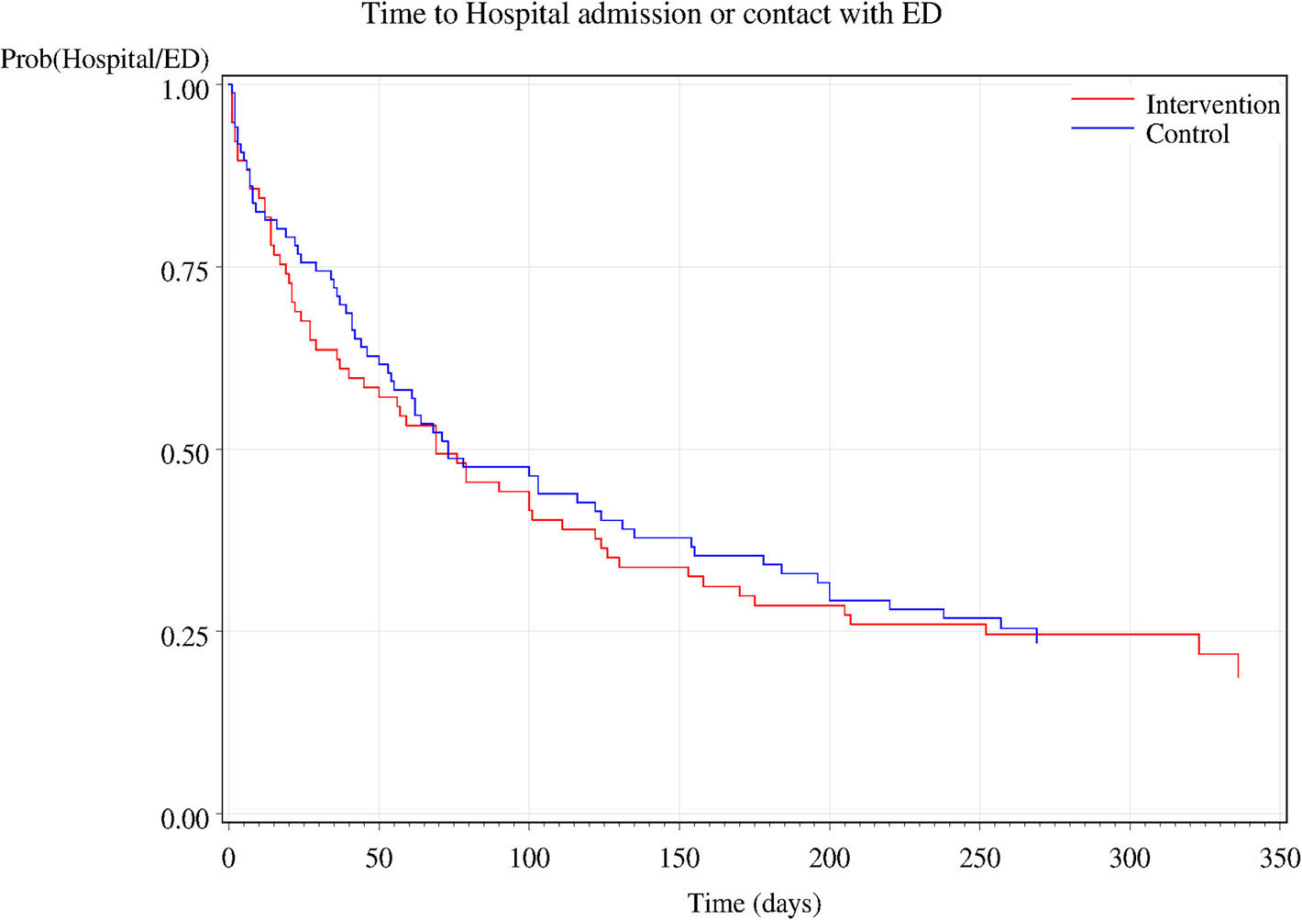
Time to hospital admission or contact with ED, according to group allocation

The proportions of patients within each group who had contact with the health service during the 3-month follow-up period were similar between groups: 43/77 (55.8%) of the intervention group vs 45/86 (52.3%) of controls (χ^2^_(1)_ = 0.20, *p* = 0.6527, Table 3; odds ratio = 1.15). The Kaplan-Meier curves for the time to first contact with the health service were drawn for the two groups (Fig. 1), and the Log-Rank test showed no statistically significant difference between curves (*p* = 0.899).

**Table 3:** Contact with the acute care system during the follow-up period (ambulance use, emergency department presentation or hospital admission)

Variable	Control	Intervention	*p*-value
Contact with health system [n/N (%)]	45/86 (52.3)	43/77 (55.8)	0.6527^#^
Hospital LOS (days) N; median (range)	42; 8.5 (1–51)	39; 8.0 (1–50)	0.8574^&^
Acute care costs (AUD$); mean (SD)	$9421 ($14,566)	$9306 ($13,734)	0.4848*

# Chi-square test

& Wilcoxon 2-sample test

*T-test following Box-Cox transformation

The total length of hospital stay (days) for the groups are shown in Table 3. These did not differ significantly between groups (*p* = 0.857), nor did box-cox transformed total acute care costs (*p* = 0.485).

### Intervention costs

Total FECH costs were on average $AUD268.28 per intervention participant, with an interquartile range from $AUD201.11 to $AUD339.08. Most of these costs related to intervention delivery ($AUD228.78 per dyad) with the remainder being training and telephone costs.

### Cost-effectiveness

An ICER of $AUD1,730.84 was calculated, indicating that intervention costs of $AUD1,730.84 were required for each additional carer reporting an improvement in preparedness to care (Table 4(a)).

**Table 4:** Cost-effectiveness of FECH according to (a) base-case analysis and (b-h) deterministic sensitivity analyses

Analysis	Branch	Cost	Incr cost	Effectiveness^a^	Incr eff	ICER^d^
a) ITT (base case)	Control	15.34	–	0.21	–	1730.84
	Intervention	283.62	268.28	0.36	0.15	
b) FACQ – Distress	Control	15.34	–	−0.09^b^	–	1788.53
	Intervention	283.62	268.28	−0.24^b^	0.15	
c) FACQ – Strain	Control	15.34	–	0.01^c^	–	1676.75
	Intervention	283.62	268.28	−0.15^c^	0.16	
d) Per-protocol analysis	Control	15.89	–	0.22	–	1431.23
	Intervention	352.53	336.34	0.45	0.24	
e) FECH costs based on 25th percentile observed during trial	Control	15.34	–	0.21	–	1297.55
	Intervention	216.46	201.12	0.36	0.15	
f) FECH costs based on 75th percentile observed within trial	Control	15.34	–	0.21	–	2187.61
	Intervention	354.42	339.08	0.36	0.15	
g) FECH effectiveness based on lower limit of 95% CI of population proportion	Control	15.34	–	0.21	–	5061.89
	Intervention	283.62	268.28	0.26	0.15	
h) FECH effectiveness based on upper limit of 95% CI of population proportion	Control	15.34	–	0.21	–	1043.89
	Intervention	283.62	268.28	0.46	0.26	

Deterministic sensitivity analyses vary from base-case as follows: (b) outcome of carer distress at Time 2; (c) outcome of carer strain at Time 3; (d) carer preparedness under per-protocol analysis; (e-f) carer preparedness at the interquartile range of FECH costs observed; (g-h) carer preparedness at the limits of the 95% CI of the proportion of carers reporting an improvement in preparedness

^a^Proportion of carers in each group reporting an improvement of at least two points on the Carer Preparedness scale to Time 2, unless otherwise stated

^b^Mean change in distress scores to Time 2

^c^Mean change in strain scores to Time 3

^d^AUD$2015

ICERs were also calculated for secondary outcomes which showed significant between group differences. On the FACQ-PC distress scale, the intervention group showed an incremental reduction 0.15 points greater than the control group to Time 2 (Table 4(b)), resulting in an ICER of $AUD1,788.53. On the FACQ-PC Strain scale the intervention group showed an incremental reduction 0.16 points greater than the control group to Time 3 (Table 4(c)) resulting in an ICER of $AUD1,676.75.

### Deterministic sensitivity analysis

In the per-protocol analysis (Table 4(d)) the increased incremental effectiveness outweighed an increase in incremental cost (caused by the costs of non-completers in the intervention group being allocated to completers) and the ICER reduced to $AUD1,431.23. If all participants had intervention costs equivalent to the 25th percentile (Table 4(e)), assuming effectiveness was unchanged, the ICER was reduced to $AUD1,297.55. If all participants had intervention costs equivalent to the 75th percentile (Table 4(f)) the ICER would be $AUD2,187.61. Table 4 (g) and (h) present ICERs for which intervention effectiveness is based on the lower and upper limits of the 95% CI of the estimated population proportion showing improvement under the intervention. At the lower limit of the confidence interval an estimated 26% would show an improvement in preparedness to care, resulting in an ICER of $AUD5,061.89, while at the upper limit 46% would show an improvement, resulting in an ICER of $AUD1,043.89.

### Probabilistic sensitivity analysis

**Fig. 2:**
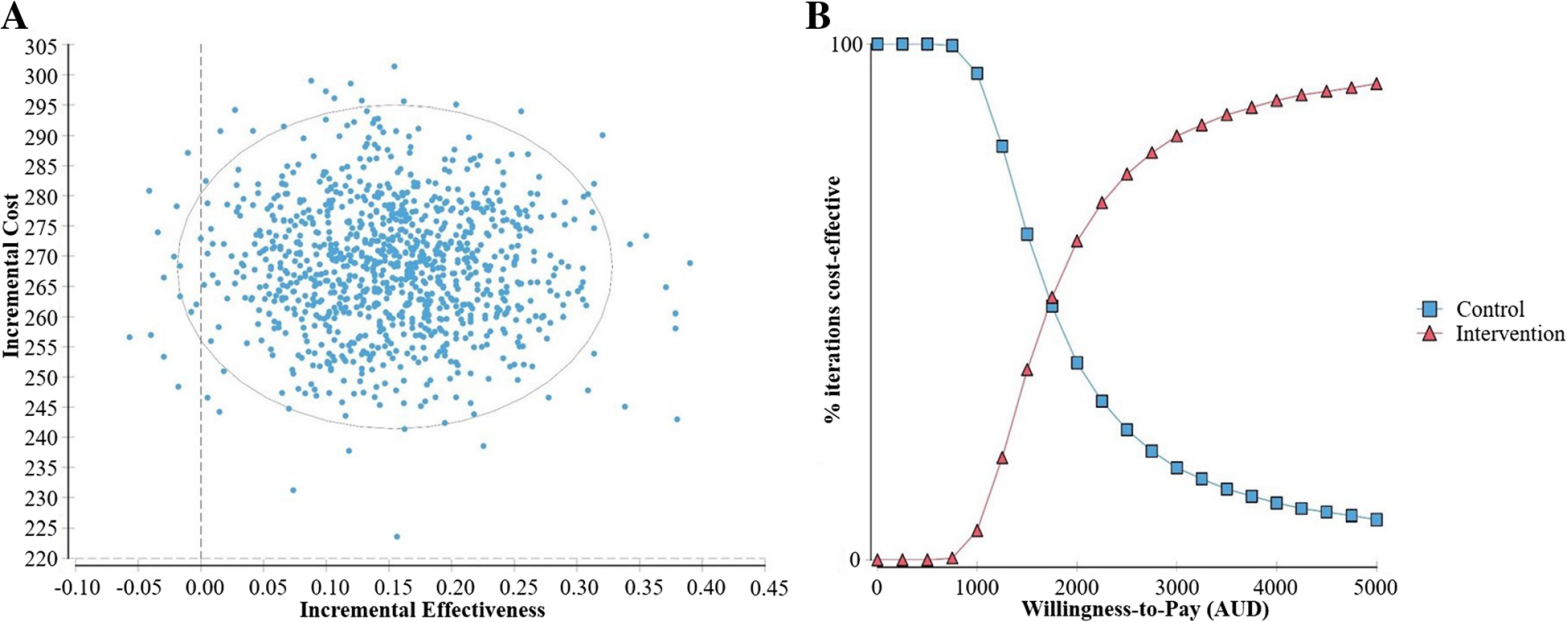
Probabilistic sensitivity analysis results including incremental cost-effectiveness scatterplot and iterations cost-effective at different willingness-to-pay values. Legend: Probabilistic Sensitivity Analysis run with 1000 iterations. Incremental cost-effectiveness scatter plot (**a**) displays incremental costs and effectiveness of intervention compared to usual care for each iteration; the oval captures 95% of iterations. Part (**b**) displays the % of iterations for which control or intervention conditions were more cost-effective, at willingness-to-pay values (values a funder may be willing to pay for each carer reporting a two-point improvement in preparedness to care) from $AUD0 to $AUD5,000

Results of PSA showed that FECH was more effective than usual care in over 98% of iterations and more costly in all cases (Fig. 2(a)). This analysis also shows that if a funder was willing to pay $AUD1,000 per carer reporting improved preparedness to care, there was a 94% probability that usual care was cost-effective, while at a willingness to pay of $AUD4,250 there was a 90% probability that FECH was cost-effective (Fig. 2(b)).

## Discussion

In this study we aimed to examine whether positive outcomes for carers resulting from FECH led to changes in the use and costs of hospital and ambulance services by patients, and to assess the cost-effectiveness of the intervention. We hypothesised that the intervention would reduce the use of health services by patients, partially offsetting the intervention costs. The FECH intervention was found to lead to improvements in preparedness to care, at a cost of $AUD1,731 for each additional caregiver reporting an improvement in preparedness to care at Time 2. There was a cost of $AUD1,789 for each additional one-point improvement in caregiver distress and $AUD1,677 for each additional one point improvement in caregiver strain. Improvements on each of these outcomes were statistically significant, as reported previously [8]. It is important to note that there is overlap between these outcomes, as each dollar invested contributes to multiple caregiver benefits. The outcomes for caregivers did not, however, translate to changes in patient use of hospital services in the three months following recruitment.

In interpreting cost-effectiveness figures it is important to consider the potential “willingness to pay” for the outcome in question [12]. In this case willingness to pay would refer to the amount the funder (i.e. the West Australian Department of Health, reflecting the perspective of the analysis) would be willing to pay for each additional caregiver reporting an improvement in preparedness to care. There is currently no guidance available as to what the funder, or anyone else, may be willing to pay for this outcome. Furthermore, we are unaware of interventions of any type which have used the preparedness for caregiving scale and reported on cost-effectiveness against which we could compare these findings.

There is a small number of published cost-effectiveness studies comparable to the FECH trial in terms of intervention aims, which focus on caregivers of people with dementia. One study, conducted in the Netherlands, trialled an intervention in which caregivers of people with dementia attended a series of sessions including counselling and family meetings individualized to meet the caregiver’s needs which aimed to offer psycho-education, teach problem-solving techniques and mobilise family networks to provide support [31]. A similar study conducted in the UK assessed an intervention of eight face to face therapy sessions delivered by specially trained health professionals for caregivers of people with dementia, with a focus on caregiver coping [32]. While the UK study reported an ICER of £189 for each carer reporting an improvement in anxiety/depressive symptoms in excess of the MCID on the scale used, the Netherlands study reported an ICER of €9271 for each incidence of major caregiver depression/anxiety avoided. The current study produced ICERs for caregiver preparedness to care, caregiver strain and caregiver distress which fell between the ICERs reported for improvements in depression/anxiety in these studies, though the different populations assessed, different health systems in which the studies were conducted and different outcomes assessed may limit comparability.

It is likely that the effectiveness and cost-effectiveness of the FECH intervention would differ across populations. Previous research into family caregivers of palliative patients found that caregivers who were less vulnerable at baseline did not benefit from a psychoeducational intervention aimed at improving preparedness for caregiving [33]. Although the intervention and patient population differed from the current study, similar issues could impact on the observed effectiveness of FECH, considering that the participating family caregivers tended to be well educated and may have had relatively few support needs [8]. A targeting of the intervention at those caregivers with greater needs may result in increased effectiveness. Of course, this may also result in higher intervention costs if additional nurse time is required in supporting such caregivers.

In this trial cost-effectiveness was improved in the per-protocol analysis compared to the LOCF “base case”. It is possible that outside of a trial setting where caregivers would not be expected to complete lengthy assessment tools in addition to the time involved participating in the intervention, follow-up and hence effectiveness may improve. Regardless, the difference in cost-effectiveness between the LOCF and per-protocol analyses suggests that targeting the intervention towards caregivers least likely to withdraw may be an essential part of making this intervention as cost-effective as possible.

We found that patient use of hospital, ED and ambulance services did not differ between groups. This may well reflect that preparedness to care, caregiver distress and caregiver strain do not impact on the health of patients, or at least on their need for hospital and ambulance services, within the brief follow-up period considered during this study. Even if caregiver preparedness does impact on need for these services, it is just one of many factors influencing hospital use. Variation introduced by other factors (e.g. patient condition, distance to services, socioeconomic status) would have the effect of making any between-group differences resulting from changes in caregiver preparedness more difficult to identify, given this study was powered for the preparedness to care outcome. Additionally we did not examine the use of primary care, community health, hospital outpatient or other services which may also be accessed by patients and caregivers facing difficulty. Given the high acute care costs across both groups within the three month follow-up, even a modest reduction as a result of FECH could make a substantial difference to cost-effectiveness. Further research, powered for health service use outcomes, would provide a vastly improved estimate of cost-effectiveness.

The intervention was found not to impact on caregiver self-rated physical or mental health to either follow-up. It is counterintuitive that the intervention’s effects on preparedness to care, caregiver strain and caregiver distress were not reflected in changes in self-rated health. It may be the case that the study, being powered to detect changes in preparedness to care, was underpowered to detect changes in self-rated health where there are likely to be many additional sources of variation present. It is also intuitive that changes in caregiver strain, caregiver distress and other outcomes may take time to filter through to caregiver health, while this study had a relatively short follow-up. In a similar CSNAT trial in community palliative care, while the intervention was associated with a significant reduction in the FACQ-PC caregiver strain, the differences in SF12v2 scores were not significant [34]. The authors postulated that this result may be due to SF12v2 not accurately capturing the outcome for the study within the short time period of the intervention, or that the intervention had limited effect in this case.

The FECH nurse recorded detailed notes on the time spent working with each caregiver during the trial, and as such we can be confident in the accuracy and completeness of intervention costs recorded here. It is worth noting here that undertaking such interventions in a research setting rather than a routine practice setting is more time consuming and more burdensome on service providers, as already reported in a similar trial [34, 35]. Therefore total costs would be reduced if and when this intervention is integrated in standard practice. Administrative data were accessed to ascertain the use of hospital, ED and ambulance services by each patient, hence these comparisons are not impacted by recall bias. Groups were well balanced at baseline on all demographic and outcome measures assessed. Although loss to follow-up differed between groups, we were able to perform cost effectiveness analysis dealing with missing data through two different methods to understand the possible impact of this on findings.

The analyses reported here have several important limitations. Firstly, the perspective of the analysis may impact on generalisability. This study focussed on hospital costs because these costs are major cost drivers [36] and are compounded by the adverse events experienced by older people in the hospital setting [37], meaning that there is a significant imperative to minimise hospitalisation for older people to minimise both costs and suffering. We were also unable to consider any impacts on patient or caregiver out-of-pocket costs. The trial had a relatively short follow-up, and it is possible that changes in some outcomes may take longer to manifest. The health conditions experienced by older people in many cases are not curable and are characterised by a gradual deterioration or repeated exacerbations [38]. As such, an intervention aimed at improving preparedness to care would ideally have a lasting effect. A future study with a longer follow-up would provide better indications of the extent to which supporting carers can help to maintain an older person at home following discharge.

Additionally, the trial was powered to detect changes in the primary outcome of caregiver preparedness, and may have been underpowered to detect changes in health service use, which may substantially impact on cost-effectiveness. Finally, some participants failed to complete all time points so missing data may impact on results here. We reported results using the conservative LOCF approach, and repeated key analysis using only data from completers (“per-protocol”) as a sensitivity analysis to better understand the impact of missing data. The choice of method did not impact on the significance of changes in preparedness at either follow-up, though the positive effect of the intervention on preparedness to care at Time 2 was greater under the per-protocol analysis. The LOCF therefore may have provided conservative estimates of cost-effectiveness.

## Conclusions

The FECH program resulted in improved preparedness to care, reduced caregiver strain and caregiver distress, although this did not translate to improvements in caregiver’s self-rated health nor in reduced use of acute care services by patients. However, this study has provided thus far the scientific/evidence-based justification needed for such person centred healthcare interventions.

This is the first work to report cost-effectiveness of telephone-based intervention to support caregivers of older people discharged from hospital, and has laid the analytic foundation for a robust economic justification when future similar studies have a larger sample size and a longer follow-up period. Both types of justifications, scientific/evidence and economic are essential to drive such person-centred healthcare interventions closer to being translated into practice and policy [39].

## Abbreviations

CSNAT: Caregiver Support Needs Assessment Tool
ED: Emergency Department
FACQ-PC: Family Appraisal of Caregiving Questionnaire for Palliative Care
FECH: Further Enabling Care at Home
GDP: Gross Domestic Product
ICER: Incremental Cost-Effectiveness Ratio
LOCF: Last Observation Carried Forward
MAU: Medical Assessment Unit
MCID: Minimum Clinically Important Difference
PSA: Probabilistic Sensitivity Analysis

## References

[1] Quiñones AR, Markwardt S, Botoseneanu A (2016). Multimorbidity combinations and disability in older adults. J Gerontol A Biol Sci Med Sci.

[2] Barrett P, Hale B, Butler M (2014). Caring for Older People. Family care and social capital: transitions in informal care.

[3] Deloitte Access Economics: The economic value of informal care in Australia in 2015. In*.* ACT: Deloitte Access Economics; 2015.

[4] Zehner Ourada VE, Walker AJ (2014). A comparison of physical health outcomes for caregiving parents and caregiving adult children. Fam Relat.

[5] Reed RL, Isherwood L, Ben-Tovim D (2015). Why do older people with multi-morbidity experience unplanned hospital admissions from the community: a root cause analysis. BMC Health Serv Res.

[6] Slayter S, Toye C, Popescu A, Young J, Matthews A, Hill A, Williamson DJ (2013). Early re-presentation to hospital after discharge from an acute medical unit: perspectives of older patients, their family caregivers and health professionals. J Clin Nurs.

[7] Whittamore KH, Goldberg SE, Bradshaw LE, Harwood RH (2014). The medical crises in older people study group: factors associated with family caregiver dissatisfaction with acute Hospital Care of Older Cognitively Impaired Relatives. J Am Geriatr Soc.

[8] Toye C, Parsons R, Slatyer S, Aoun SM, Moorin R, Osseiran-Moisson R, Hill KD (2016). Outcomes for family carers of a nurse-delivered hospital discharge intervention for older people (the further enabling Care at Home Program): single blind randomised controlled trial. Int J Nurs Stud.

[9] Bakas T, Austin JK, Habermann B, Jessup NM, McLennon SM, Mitchell PH, Morrison G, Yang Z, Stump TE, Weaver MT (2015). Telephone assessment and skill-building kit for stroke caregivers: a randomized controlled clinical trial. Stroke.

[10] Jackson D, Roberts R, Wu ML, Ford R, Doyle C (2015). A systematic review of the effect of telephone, internet or combined support for carers of people living with Alzheimers, vascular or mixed dementia in the community. Arch Gerontol Geriatr.

[11] Health Expenditure [http://www.oecd.org/health/health-expenditure.htm]. Accessed 11 Dec 2017.

[12] Drummond MF, Stoddart GL, Torrance GW (1987). Methods for the economic evaluation of health care Programmes.

[13] Toye C, Moorin R, Slatyer S, Aoun SM, Parsons R, Hegney D, Maher S, Hill KD (2015). Protocol for a randomised controlled trial of an outreach support program for family carers of older people discharged from hospital. BMC Geriatr.

[14] Husereau D, Drummond M, Petrou S, Carswell C, Moher D, Greenberg D, Augustovski F, Briggs A, Mauskopf J, Loder E: Consolidated health economic evaluation reporting standards (CHEERS) statement. Br Med J 2013, 346(f1049). 10.1136/bmj.f1049 23529982

[15] Archbold PG, Stewart BJ, Greenlick MR, Harvath T (1990). Mutuality and preparedness as predictors of caregiver role strain. Research in Nursing & Health.

[16] Department of Health and Ageing: 2007–08 Annu Rep for the Department of Health and Ageing. In*.* Edited by Ageing DoHa. Canberra: Australian Government; 2008.

[17] Aggar C, Ronaldson S, Cameron ID (2011). Self-esteem in carers of frail older people: resentment predicts anxiety and depression. Aging Ment Health.

[18] Ewing G, Brundle C, Payne S, Grande G (2013). The Carer support needs assessment tool (CSNAT) for use in palliative and end-of-life care at home: a validation study. J Pain Symptom Manag.

[19] Ewing G, Grande G (2013). The Carer support needs assessment tool (CSNAT) for end-of-life care practice at home: a qualitative study. Palliat Med.

[20] Aoun SM, Stegmann R, Slatyer S, Hill KD, Parsons R, Moorin R, Bronson M, Walsh D, Toye C (2018). Hospital postdischarge intervention trialled with family caregivers of older people in Western Australia: potential translation into practice. BMJ Open.

[21] National Health and Medical Research Council (NHMRC) (2015). National statement on ethical conduct in human research 2007 (updated May 2015).

[22] Cooper B, Kinsella GJ, Picton C (2006). Development and initial validation of a family appraisal of caregiving questionnaire for palliative care. Psycho-oncology.

[23] Ware JE, Kosinski MM, Keller S (1996). A 12-item short-form health survey: construction of scales and preliminary tests of reliability and validity. Med Care.

[24] Holman CDJ, Bass AJ, Rouse LL, Hobbs MST (1999). Population-based linkage of health records in Western Australia: development of a health services research linked database. Aust N Z J Public Health.

[25] Authority IHP (2016). Australian public hospitals cost report 2014–15 round 19.

[26] Metro Ambulance Fees [http://www.stjohnambulance.com.au/ambulance-and-health-services/metro-ambulance-service/metro-ambulance-fees]. Accessed 10 Feb 2015.

[27] Historical Data [https://www.rba.gov.au/statistics/historical-data.html]. Accessed 7 Nov 2017.

[28] corp IBM (2013). SPSS statistics for windows. In*.*, 22.0 edn. Armonk.

[29] TreeAge Software Inc (2017). TreeAge Pro 2017.

[30] Zweifel P, Breyer F, Kifmann M (1997). Health economics.

[31] Joling KJ, Bosmans JE, van Marwijk HWJ, van der Horst HE, Scheltens P, MacNeil Vroomen JL, van Hout HPJ (2013). The cost-effectiveness of a family meetings intervention to prevent depression and anxiety in family caregivers of patients with dementia: a randomised trial. Trials.

[32] Knapp M, King D, Romeo R, Schehl B, Barber J, Griffin M, et al. Cost-effectiveness of a manual based coping strategy programme in promoting the mental health of family caregivers of people with dementia (the START (STrAtegies for RelaTives) study): a pragmatic randomised controlled trial. BMJ. 2013;347. 10.1136/bmj.f6342 PMC3808080 24162943

[33] Holm M, Arestedt K, Carlander I, Wengstrom Y, Ohlen J, Alvariza A (2017). Characteristics of the family caregivers who did not benefit from a successful psychoeducational group intervention during palliative Cancer care: a prospective correlational study. Cancer Nurs.

[34] Aoun SM, Grande G, Howting D, Deas K, Toye C, Troueung L, et al. The impact of the Carer support needs assessment tool (CSNAT) in community palliative care using a stepped wedge cluster trial. PLoS One. 2015;10(4). 10.1371/journal.pone.0123012 PMC4388632 25849348

[35] Aoun S, Toye C, Deas K, Howting D, Ewing G, Grande G, Stajduhar K (2015). Enabling a family caregiver-led assessment of support needs in home-based palliative care: potential translation into practice. Palliat Med.

[36] Australian Institute of Health and Welfare: Australia's health 2016. In Canberra:AIHW; 2016.

[37] Bail K, Goss J, Draper B, Berry H, Karmel R, Gibson D (2015). The cost of hospital-acquired complications for older people with and without dementia; a retrospective cohort study. BMC Health Serv Res.

[38] Lunney J, Lynn J, Foley D, Lipson S, Guralnik J (2003). Patterns of functional decline at the end of life. J Am Med Assoc.

[39] Miles A, Asbridge J (2017). Person-Centred healthcare - moving from rhetoric to methods, through implementation to outcomes. Eur. J. Pers. Cent. Healthc.

